# Crystal structure of ethyl 2-({[(4*Z*)-3,5-dioxo-1-phenyl­pyrazolidin-4-yl­idene]meth­yl}amino)­acetate

**DOI:** 10.1107/S1600536814016766

**Published:** 2014-08-01

**Authors:** Shaaban K. Mohamed, Mehmet Akkurt, Joel T. Mague, Eman A. Ahmed, Mustafa R. Albayati

**Affiliations:** a Chemistry and Environmental Division, Manchester Metropolitan University, Manchester M1 5GD, England; b Chemistry Department, Faculty of Science, Minia University, 61519 El-Minia, Egypt; c Department of Physics, Faculty of Sciences, Erciyes University, 38039 Kayseri, Turkey; d Department of Chemistry, Tulane University, New Orleans, LA 70118, USA; e Chemistry Department, Faculty of Science, Sohag University, 82524 Sohag, Egypt; f Kirkuk University, College of Science, Department of Chemistry, Kirkuk, Iraq

**Keywords:** crystal structure, hydrogen bonding, hydrogen-bonded dimers, pyrazolidine-3,5-dione, amino­acetic acid ester

## Abstract

The title compound, C_14_H_15_N_3_O_4_, is nearly planar, the dihedral angle between the planes of the phenyl and pyrazolidine rings being 1.13 (7) Å, and that between the plane of the pyrazolidine ring and the mean plane of the side chain [C—N—C–C(=O)—O; r.m.s. deviation = 0.024 Å] being 2.52 (7)°. This is due in large part to the presence of the intra­molecular N—H⋯O and C—H⋯O hydrogen bonds. In the crystal, pairwise N—H⋯O hydrogen bonds form inversion dimers, which are further associated into layers, lying very close to plane (-120), *via* pairwise C—H⋯O hydrogen bonds. The layers are then weakly connected through C—H⋯O hydrogen bonds, forming a three-dimensional structure.

## Related literature   

For the synthesis of compounds containing the pyrazolidinone nucleus and their biological activity, see: Ismail *et al.* (2012 ▶); Khodairy (2007 ▶); Khloya *et al.* (2013 ▶). For biologically active synthetic heterocyclic compounds containing the pyrazol-5(4*H*)-one core scaffold and displaying some inter­esting pharmaceutical properties, see: Uramaru *et al.* (2010 ▶) for analgesic; Thaker *et al.* (2011 ▶) and Chande *et al.* (2007 ▶) for anti­microbial; Mariappan *et al.* (2010 ▶) and Nishikimi *et al.* (2012 ▶) for anti-inflammatory; Chen *et al.* (2012 ▶) for cyto­toxicity.
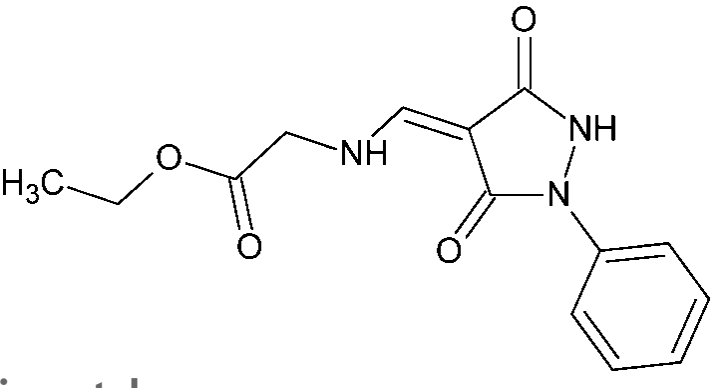


## Experimental   

### Crystal data  

C_14_H_15_N_3_O_4_
*M* *_r_* = 289.29
Triclinic, 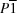

*a* = 5.4984 (1) Å
*b* = 7.3585 (2) Å
*c* = 16.6265 (4) Å
α = 91.3290 (9)°
β = 97.325 (1)°
γ = 99.562 (1)°
*V* = 657.27 (3) Å^3^
*Z* = 2
Cu *K*α radiation
μ = 0.91 mm^−1^
*T* = 100 K
0.27 × 0.09 × 0.04 mm

### Data collection  

Bruker D8 VENTURE PHOTON 100 CMOS diffractometer
Absorption correction: multi-scan (*SADABS*; Bruker, 2013 ▶) *T*_min_ = 0.91, *T*_max_ = 0.96
5108 measured reflections
2450 independent reflections
2171 reflections with *I* > 2σ(*I*)
*R*_int_ = 0.019

### Refinement  

*R*[*F* ^2^ > 2σ(*F* ^2^)] = 0.033
*wR*(*F* ^2^) = 0.089
*S* = 1.02
2450 reflections
199 parameters
H atoms treated by a mixture of independent and constrained refinement
Δρ_max_ = 0.22 e Å^−3^
Δρ_min_ = −0.19 e Å^−3^

### 

Data collection: *APEX2* (Bruker, 2013 ▶); cell refinement: *SAINT* (Bruker, 2013 ▶); data reduction: *SAINT*; program(s) used to solve structure: *SHELXT* (Sheldrick, 2008 ▶); program(s) used to refine structure: *SHELXL2014* (Sheldrick, 2008 ▶); molecular graphics: *DIAMOND* (Brandenburg & Putz, 2012 ▶); software used to prepare material for publication: *SHELXTL* (Sheldrick, 2008 ▶).

## Supplementary Material

Crystal structure: contains datablock(s) global, I. DOI: 10.1107/S1600536814016766/su2759sup1.cif

Structure factors: contains datablock(s) I. DOI: 10.1107/S1600536814016766/su2759Isup2.hkl

Supporting information file. DOI: 10.1107/S1600536814016766/su2759Isup3.cml

**Fig. 1.**
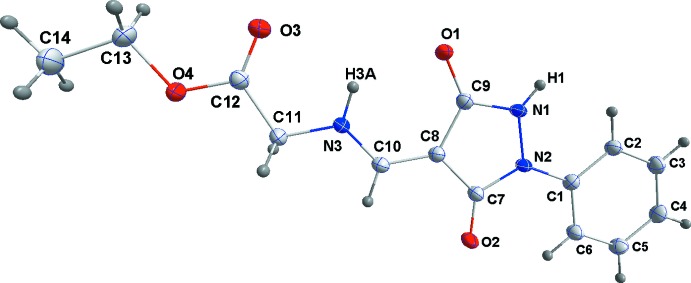
The molecular structure of the title molecule, with atom labelling. Displacement ellipsoids are drawn at the 50% probability level.

**Fig. 2.**
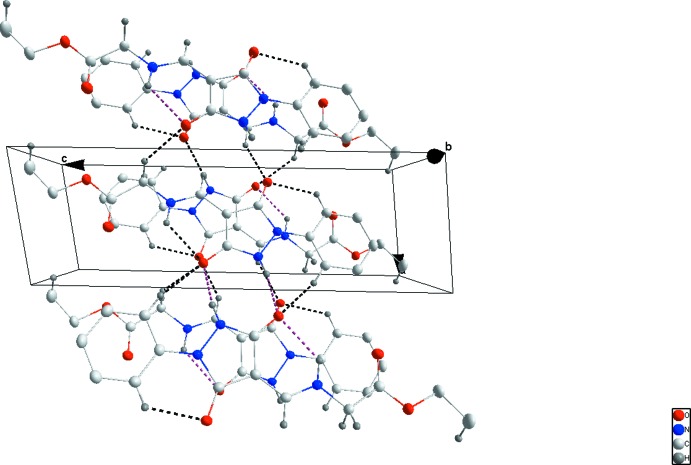
The crystal packing viewed along the b axis of the title compound. N—H···O and C—H···O hydrogen bonds are shown, respectively, as purple and black dotted lines (see Table 1 for details).

. DOI: 10.1107/S1600536814016766/su2759fig1.tif
The mol­ecular structure of the title mol­ecule, with atom labelling. Displacement ellipsoids are drawn at the 50% probability level.

b . DOI: 10.1107/S1600536814016766/su2759fig2.tif
The crystal packing viewed along the *b* axis of the title compound. N—H⋯O and C—H⋯O hydrogen bonds are shown, respectively, as purple and black dotted lines (see Table 1 for details).

**Fig. 3.**
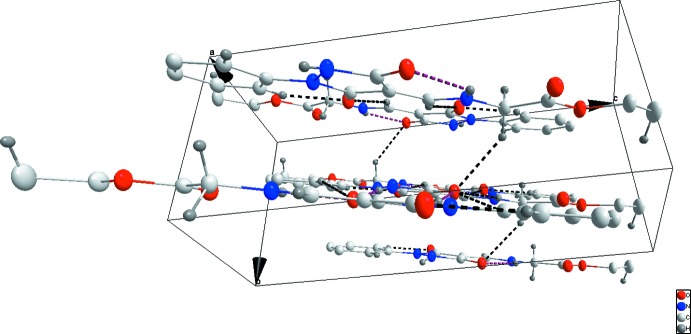
The crystal packing of the title compound, showing the layer structure and the weak C—H···O interlayer hydrogen bonds (black dotted lines; see Table 1 for details).

. DOI: 10.1107/S1600536814016766/su2759fig3.tif
The crystal packing of the title compound, showing the layer structure and the weak C—H⋯O inter­layer hydrogen bonds (black dotted lines; see Table 1 for details).

CCDC reference: 1015152

Additional supporting information:  crystallographic information; 3D view; checkCIF report

## Figures and Tables

**Table 1:** Hydrogen-bond geometry (Å, °)

*D*—H⋯*A*	*D*—H	H⋯*A*	*D*⋯*A*	*D*—H⋯*A*
N3—H3*A*⋯O1	0.914 (18)	2.250 (17)	2.9062 (14)	128.3 (13)
C6—H6⋯O2	0.95	2.23	2.8833 (16)	126
N1—H1⋯O1^i^	0.88 (2)	1.89 (2)	2.7573 (14)	170.7 (16)
C2—H2⋯O1^i^	0.95	2.36	3.2777 (15)	162
C10—H10⋯O2^ii^	0.95	2.28	3.1268 (16)	148
C11—H11*A*⋯O2^ii^	0.99	2.58	3.1498 (15)	117
C11—H11*B*⋯O1^iii^	0.99	2.51	3.3386 (15)	142

Symmetry codes: (i) 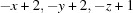; (ii) 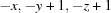; (iii) 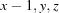.

## supplementary crystallographic information

### S1. Experimental

A mixture of 1 mmol (231 mg) of (4*Z*)-4-[(dimethylamino)methylene]-1-phenylpyrazolidine-3,5-dione and 1 mmol (140 mg) of ethyl aminoacetate hydrochloride and few drops of triethylamine (TEA) as a catalyst in 30 ml 1,4-dioxane was refluxed for 6 h. On cooling the solid product deposited, was filtered off, washed with cold ethanol and dried under vacuum. Crystals of the title compound were obtained as yellow needles by recrystallization of the crude product from dimethyl sulfoxide; *M*.p. 489–491 K.

### S2. Refinement

The N-bound H atoms were located in a Fourier difference map and freely refined. The C-bound H atoms were placed in calculated positions and treated as riding atoms: C—H = 0.95 - 0.99 Å with U_iso_(H) = 1.5U_eq_(C-methyl) and = 1.2U_eq_(C) for other H atoms. The crystal did not diffract well, hence the lower than desirable value for θ_full_.

### Crystal data

**Table:**

C_14_H_15_N_3_O_4_	*Z* = 2
*M**_r_* = 289.29	*F*(000) = 304
Triclinic, *P*1	*D*_x_ = 1.462 Mg m^−^^3^
*a* = 5.4984 (1) Å	Cu *K*α radiation, λ = 1.54178 Å
*b* = 7.3585 (2) Å	Cell parameters from 3908 reflections
*c* = 16.6265 (4) Å	θ = 2.7–72.2°
α = 91.3290 (9)°	µ = 0.91 mm^−^^1^
β = 97.325 (1)°	*T* = 100 K
γ = 99.562 (1)°	Needle, yellow
*V* = 657.27 (3) Å^3^	0.27 × 0.09 × 0.04 mm

### Data collection

**Table:**

Bruker D8 VENTURE PHOTON 100 CMOS diffractometer	2450 independent reflections
Radiation source: INCOATEC IµS micro-focus source	2171 reflections with *I* > 2σ(*I*)
Mirror monochromator	*R*_int_ = 0.019
Detector resolution: 10.4167 pixels mm^-1^	θ_max_ = 72.2°, θ_min_ = 5.4°
ω scans	*h* = −6→6
Absorption correction: multi-scan (*SADABS*; Bruker, 2013)	*k* = −9→8
*T*_min_ = 0.91, *T*_max_ = 0.96	*l* = −20→20
5108 measured reflections	

### Refinement

**Table:**

Refinement on *F*^2^	Primary atom site location: structure-invariant direct methods
Least-squares matrix: full	Secondary atom site location: difference Fourier map
*R*[*F*^2^ > 2σ(*F*^2^)] = 0.033	Hydrogen site location: mixed
*wR*(*F*^2^) = 0.089	H atoms treated by a mixture of independent and constrained refinement
*S* = 1.02	*w* = 1/[σ^2^(*F*_o_^2^) + (0.0471*P*)^2^ + 0.2143*P*] where *P* = (*F*_o_^2^ + 2*F*_c_^2^)/3
2450 reflections	(Δ/σ)_max_ < 0.001
199 parameters	Δρ_max_ = 0.22 e Å^−^^3^
0 restraints	Δρ_min_ = −0.19 e Å^−^^3^

### Special details

**Table:**

Geometry. All e.s.d.'s (except the e.s.d. in the dihedral angle between two l.s. planes) are estimated using the full covariance matrix. The cell e.s.d.'s are taken into account individually in the estimation of e.s.d.'s in distances, angles and torsion angles; correlations between e.s.d.'s in cell parameters are only used when they are defined by crystal symmetry. An approximate (isotropic) treatment of cell e.s.d.'s is used for estimating e.s.d.'s involving l.s. planes.
Refinement. Refinement of *F*^2^ against ALL reflections. The weighted *R*-factor *wR* and goodness of fit *S* are based on *F*^2^, conventional *R*-factors *R* are based on *F*, with *F* set to zero for negative *F*^2^. The threshold expression of *F*^2^ > σ(*F*^2^) is used only for calculating *R*-factors(gt) *etc*. and is not relevant to the choice of reflections for refinement. *R*-factors based on *F*^2^ are statistically about twice as large as those based on *F*, and *R*- factors based on ALL data will be even larger. H-atoms attached to carbon were placed in calculated positions (C—H = 0.95 - 0.99 Å) and included as riding contributions with isotropic displacement parameters 1.2 - 1.5 times those of the attached carbon atoms. H-atoms attached to nitrogen were refined independently.

### Fractional atomic coordinates and isotropic or equivalent isotropic displacement parameters (Å^2^)

**Table:**

	*x*	*y*	*z*	*U*_iso_*/*U*_eq_	
O1	0.81993 (17)	0.91924 (12)	0.58190 (5)	0.0208 (2)	
O2	0.18424 (17)	0.56447 (13)	0.39606 (5)	0.0232 (2)	
O3	0.56220 (17)	0.89351 (13)	0.80229 (5)	0.0238 (2)	
O4	0.20347 (17)	0.77726 (12)	0.84951 (5)	0.0214 (2)	
N1	0.7714 (2)	0.82957 (15)	0.44586 (6)	0.0193 (2)	
N2	0.5788 (2)	0.73223 (15)	0.38878 (6)	0.0182 (2)	
N3	0.3705 (2)	0.76426 (15)	0.64837 (6)	0.0185 (2)	
C1	0.6132 (2)	0.71090 (16)	0.30668 (7)	0.0171 (3)	
C2	0.8342 (2)	0.79219 (17)	0.27959 (7)	0.0187 (3)	
H2	0.9640	0.8617	0.3167	0.022*	
C3	0.8642 (3)	0.77134 (18)	0.19820 (8)	0.0221 (3)	
H3	1.0142	0.8281	0.1799	0.026*	
C4	0.6776 (3)	0.66856 (18)	0.14350 (8)	0.0224 (3)	
H4	0.6992	0.6543	0.0881	0.027*	
C5	0.4589 (3)	0.58691 (18)	0.17083 (8)	0.0224 (3)	
H5	0.3308	0.5160	0.1336	0.027*	
C6	0.4240 (2)	0.60707 (17)	0.25156 (7)	0.0203 (3)	
H6	0.2729	0.5509	0.2693	0.024*	
C7	0.3762 (2)	0.66257 (17)	0.42850 (7)	0.0177 (3)	
C8	0.4474 (2)	0.72955 (16)	0.51201 (7)	0.0173 (3)	
C9	0.6925 (2)	0.83530 (17)	0.51974 (7)	0.0175 (3)	
C10	0.3003 (2)	0.69795 (17)	0.57301 (7)	0.0172 (3)	
H10	0.1399	0.6246	0.5602	0.021*	
C11	0.2115 (2)	0.73107 (18)	0.71157 (7)	0.0187 (3)	
H11A	0.1573	0.5967	0.7154	0.022*	
H11B	0.0614	0.7881	0.6980	0.022*	
C12	0.3509 (2)	0.81183 (17)	0.79164 (7)	0.0188 (3)	
C13	0.3112 (3)	0.84585 (19)	0.93050 (7)	0.0239 (3)	
H13A	0.3525	0.9822	0.9326	0.029*	
H13B	0.4654	0.7958	0.9472	0.029*	
C14	0.1209 (3)	0.7838 (2)	0.98568 (8)	0.0326 (3)	
H14A	−0.0333	0.8293	0.9670	0.049*	
H14B	0.1841	0.8327	1.0411	0.049*	
H14C	0.0877	0.6487	0.9850	0.049*	
H1	0.896 (4)	0.907 (3)	0.4313 (10)	0.034 (5)*	
H3A	0.525 (3)	0.834 (2)	0.6622 (10)	0.027 (4)*	

### Atomic displacement parameters (Å^2^)

**Table:**

	*U*^11^	*U*^22^	*U*^33^	*U*^12^	*U*^13^	*U*^23^
O1	0.0191 (5)	0.0237 (5)	0.0168 (4)	−0.0039 (4)	0.0017 (3)	−0.0013 (3)
O2	0.0169 (5)	0.0300 (5)	0.0190 (4)	−0.0062 (4)	0.0019 (3)	−0.0010 (4)
O3	0.0199 (5)	0.0270 (5)	0.0218 (4)	−0.0024 (4)	0.0014 (4)	−0.0012 (4)
O4	0.0214 (5)	0.0253 (5)	0.0158 (4)	−0.0016 (4)	0.0035 (3)	−0.0016 (3)
N1	0.0157 (6)	0.0226 (5)	0.0166 (5)	−0.0056 (4)	0.0023 (4)	−0.0017 (4)
N2	0.0154 (5)	0.0210 (5)	0.0158 (5)	−0.0029 (4)	0.0012 (4)	−0.0016 (4)
N3	0.0160 (6)	0.0208 (5)	0.0171 (5)	−0.0018 (4)	0.0028 (4)	0.0003 (4)
C1	0.0190 (6)	0.0162 (6)	0.0163 (6)	0.0036 (5)	0.0020 (5)	0.0012 (5)
C2	0.0182 (6)	0.0179 (6)	0.0193 (6)	0.0010 (5)	0.0023 (5)	0.0005 (5)
C3	0.0220 (7)	0.0228 (6)	0.0222 (6)	0.0034 (5)	0.0062 (5)	0.0028 (5)
C4	0.0275 (7)	0.0245 (7)	0.0161 (6)	0.0062 (6)	0.0037 (5)	0.0009 (5)
C5	0.0230 (7)	0.0236 (7)	0.0189 (6)	0.0022 (5)	−0.0005 (5)	−0.0009 (5)
C6	0.0181 (7)	0.0216 (6)	0.0197 (6)	0.0002 (5)	0.0018 (5)	0.0003 (5)
C7	0.0156 (6)	0.0179 (6)	0.0188 (6)	0.0005 (5)	0.0027 (5)	0.0017 (5)
C8	0.0168 (6)	0.0166 (6)	0.0174 (6)	0.0009 (5)	0.0011 (5)	0.0009 (5)
C9	0.0182 (6)	0.0164 (6)	0.0177 (6)	0.0016 (5)	0.0027 (5)	0.0010 (4)
C10	0.0149 (6)	0.0165 (6)	0.0189 (6)	0.0003 (5)	0.0009 (4)	0.0017 (4)
C11	0.0170 (6)	0.0212 (6)	0.0169 (6)	−0.0005 (5)	0.0036 (5)	0.0006 (5)
C12	0.0193 (7)	0.0177 (6)	0.0189 (6)	0.0022 (5)	0.0022 (5)	0.0015 (5)
C13	0.0270 (7)	0.0268 (7)	0.0157 (6)	0.0006 (6)	0.0008 (5)	−0.0021 (5)
C14	0.0382 (9)	0.0375 (8)	0.0204 (7)	−0.0017 (7)	0.0077 (6)	−0.0002 (6)

### Geometric parameters (Å, º)

**Table:**

O1—C9	1.2580 (15)	C4—C5	1.388 (2)
O2—C7	1.2288 (16)	C4—H4	0.9500
O3—C12	1.2052 (17)	C5—C6	1.3879 (18)
O4—C12	1.3378 (16)	C5—H5	0.9500
O4—C13	1.4438 (14)	C6—H6	0.9500
N1—C9	1.3557 (16)	C7—C8	1.4455 (17)
N1—N2	1.4126 (14)	C8—C10	1.3764 (18)
N1—H1	0.88 (2)	C8—C9	1.4289 (18)
N2—C7	1.3975 (17)	C10—H10	0.9500
N2—C1	1.4105 (16)	C11—C12	1.5049 (16)
N3—C10	1.3201 (16)	C11—H11A	0.9900
N3—C11	1.4503 (16)	C11—H11B	0.9900
N3—H3A	0.914 (18)	C13—C14	1.500 (2)
C1—C2	1.3958 (19)	C13—H13A	0.9900
C1—C6	1.4023 (17)	C13—H13B	0.9900
C2—C3	1.3920 (18)	C14—H14A	0.9800
C2—H2	0.9500	C14—H14B	0.9800
C3—C4	1.3873 (18)	C14—H14C	0.9800
C3—H3	0.9500		
			
C12—O4—C13	115.89 (10)	C10—C8—C9	126.25 (11)
C9—N1—N2	109.72 (10)	C10—C8—C7	125.21 (12)
C9—N1—H1	124.3 (11)	C9—C8—C7	108.54 (11)
N2—N1—H1	122.3 (11)	O1—C9—N1	124.26 (12)
C7—N2—C1	130.04 (10)	O1—C9—C8	128.51 (12)
C7—N2—N1	109.26 (10)	N1—C9—C8	107.22 (11)
C1—N2—N1	120.63 (10)	N3—C10—C8	123.39 (12)
C10—N3—C11	122.52 (11)	N3—C10—H10	118.3
C10—N3—H3A	119.8 (10)	C8—C10—H10	118.3
C11—N3—H3A	117.7 (10)	N3—C11—C12	109.81 (10)
C2—C1—C6	119.51 (11)	N3—C11—H11A	109.7
C2—C1—N2	120.64 (11)	C12—C11—H11A	109.7
C6—C1—N2	119.86 (11)	N3—C11—H11B	109.7
C3—C2—C1	119.92 (12)	C12—C11—H11B	109.7
C3—C2—H2	120.0	H11A—C11—H11B	108.2
C1—C2—H2	120.0	O3—C12—O4	125.26 (12)
C4—C3—C2	120.74 (12)	O3—C12—C11	125.64 (12)
C4—C3—H3	119.6	O4—C12—C11	109.10 (10)
C2—C3—H3	119.6	O4—C13—C14	106.97 (11)
C3—C4—C5	119.13 (12)	O4—C13—H13A	110.3
C3—C4—H4	120.4	C14—C13—H13A	110.3
C5—C4—H4	120.4	O4—C13—H13B	110.3
C4—C5—C6	121.15 (12)	C14—C13—H13B	110.3
C4—C5—H5	119.4	H13A—C13—H13B	108.6
C6—C5—H5	119.4	C13—C14—H14A	109.5
C5—C6—C1	119.55 (12)	C13—C14—H14B	109.5
C5—C6—H6	120.2	H14A—C14—H14B	109.5
C1—C6—H6	120.2	C13—C14—H14C	109.5
O2—C7—N2	124.92 (11)	H14A—C14—H14C	109.5
O2—C7—C8	129.97 (12)	H14B—C14—H14C	109.5
N2—C7—C8	105.10 (10)		
			
C9—N1—N2—C7	−4.17 (14)	N2—C7—C8—C10	178.32 (11)
C9—N1—N2—C1	178.53 (10)	O2—C7—C8—C9	178.57 (13)
C7—N2—C1—C2	−179.41 (12)	N2—C7—C8—C9	−0.82 (13)
N1—N2—C1—C2	−2.73 (17)	N2—N1—C9—O1	−176.95 (11)
C7—N2—C1—C6	0.56 (19)	N2—N1—C9—C8	3.52 (13)
N1—N2—C1—C6	177.24 (11)	C10—C8—C9—O1	−0.3 (2)
C6—C1—C2—C3	0.66 (18)	C7—C8—C9—O1	178.83 (12)
N2—C1—C2—C3	−179.37 (11)	C10—C8—C9—N1	179.20 (12)
C1—C2—C3—C4	−0.74 (19)	C7—C8—C9—N1	−1.67 (14)
C2—C3—C4—C5	0.28 (19)	C11—N3—C10—C8	179.28 (12)
C3—C4—C5—C6	0.26 (19)	C9—C8—C10—N3	0.2 (2)
C4—C5—C6—C1	−0.33 (19)	C7—C8—C10—N3	−178.81 (11)
C2—C1—C6—C5	−0.13 (19)	C10—N3—C11—C12	175.64 (11)
N2—C1—C6—C5	179.90 (11)	C13—O4—C12—O3	0.30 (18)
C1—N2—C7—O2	0.5 (2)	C13—O4—C12—C11	179.81 (10)
N1—N2—C7—O2	−176.50 (12)	N3—C11—C12—O3	0.61 (18)
C1—N2—C7—C8	179.91 (11)	N3—C11—C12—O4	−178.90 (10)
N1—N2—C7—C8	2.93 (13)	C12—O4—C13—C14	−177.08 (11)
O2—C7—C8—C10	−2.3 (2)		

### Hydrogen-bond geometry (Å, º)

**Table:**

*D*—H···*A*	*D*—H	H···*A*	*D*···*A*	*D*—H···*A*
N3—H3*A*···O1	0.914 (18)	2.250 (17)	2.9062 (14)	128.3 (13)
C6—H6···O2	0.95	2.23	2.8833 (16)	126
N1—H1···O1^i^	0.88 (2)	1.89 (2)	2.7573 (14)	170.7 (16)
C2—H2···O1^i^	0.95	2.36	3.2777 (15)	162
C10—H10···O2^ii^	0.95	2.28	3.1268 (16)	148
C11—H11*A*···O2^ii^	0.99	2.58	3.1498 (15)	117
C11—H11*B*···O1^iii^	0.99	2.51	3.3386 (15)	142

Symmetry codes: (i) −*x*+2, −*y*+2, −*z*+1; (ii) −*x*, −*y*+1, −*z*+1; (iii) *x*−1, *y*, *z*.
