# Supplementary figures and images for: Crystal structure of ethyl 2-({[(4Z)-3,5-dioxo-1-phenyl­pyrazolidin-4-yl­idene]meth­yl}amino)­acetate
Source: Acta Crystallogr Sect E Struct Rep Online. 2014 Aug 1;70(Pt 9):o938–9. doi: 10.1107/S1600536814016766 (PMC4186195; doi:10.1107/S1600536814016766)

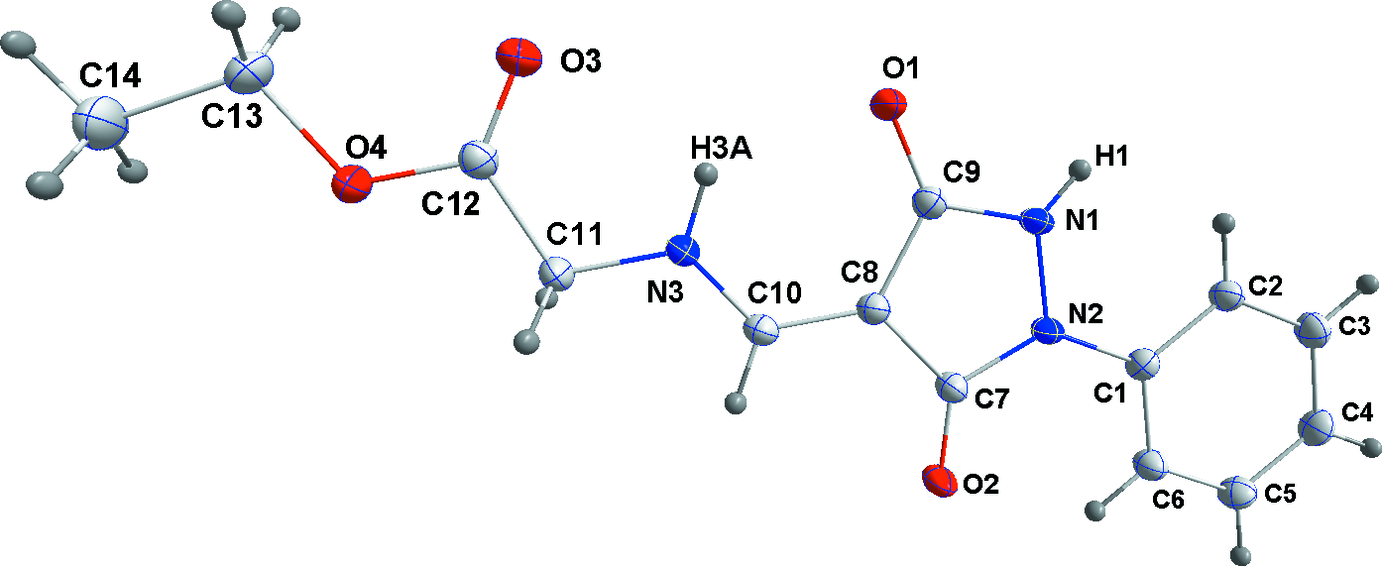

Supplement: Supplementary file 4 [file e-70-0o938-fig1.tif]

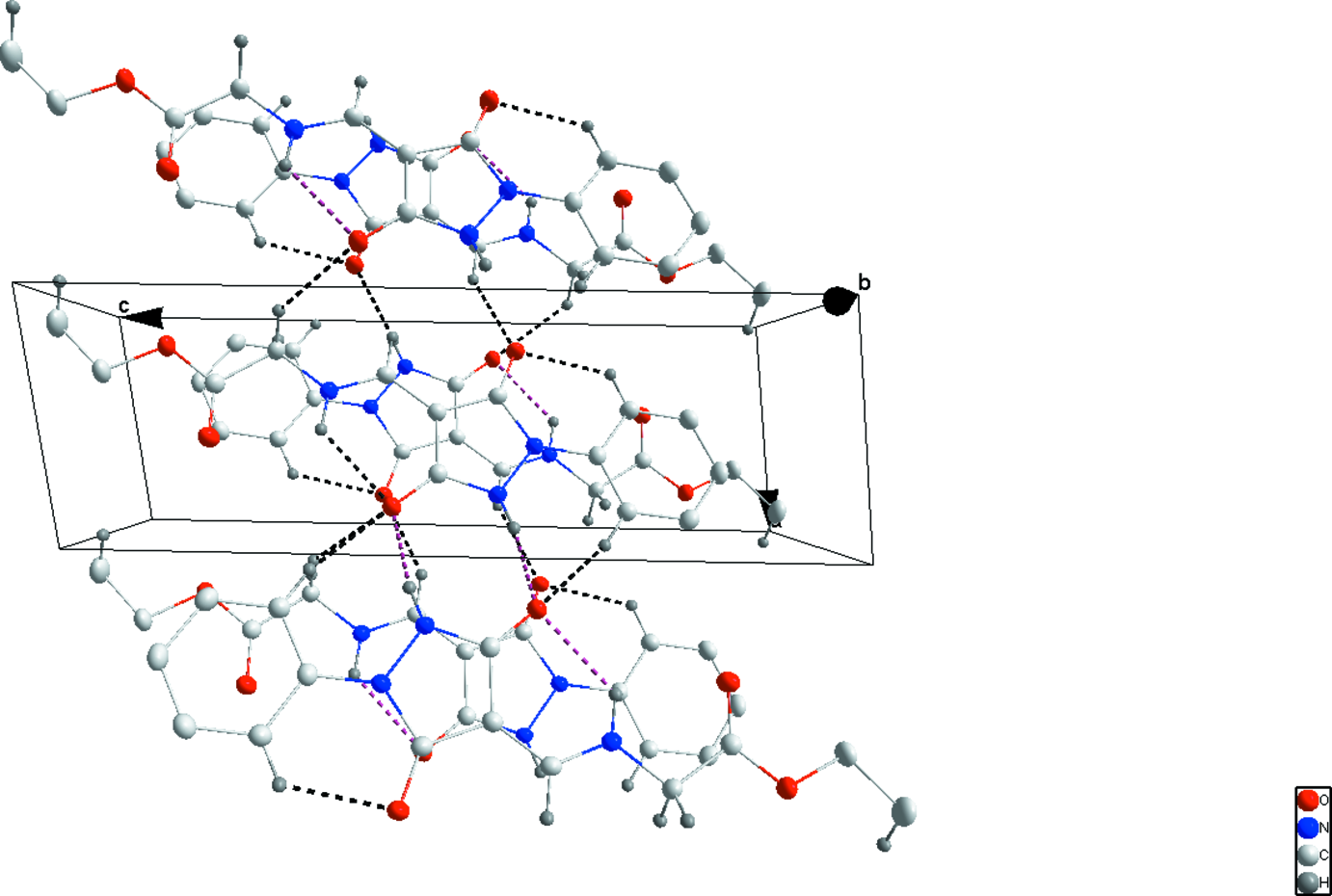

Supplement: Supplementary file 5 [file e-70-0o938-fig2.tif]

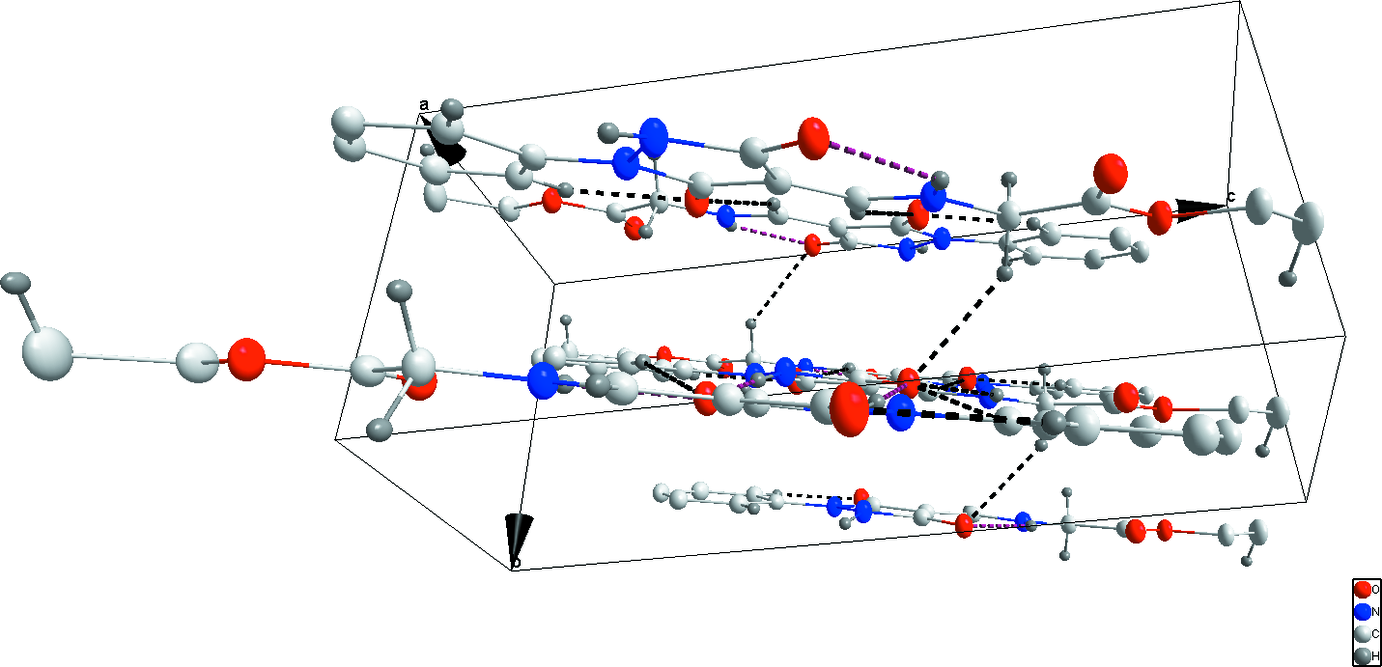

Supplement: Supplementary file 6 [file e-70-0o938-fig3.tif]
